# Radiation induced delayed transverse myelitis and neurological deficit at tertiary care center

**DOI:** 10.1016/j.amsu.2021.102728

**Published:** 2021-08-18

**Authors:** Yousef S. Abuzneid, Hamdi Al-Janazreh, Motasem Haif, Shahd T. Idais, Baraa Asakrah, Sufana M. Ajwa, Shifa Sarahneh, Hani Abdeen

**Affiliations:** a Al-Quds University Faculty of Medicine, Jerusalem, State of Palestine; b Hematology Department and Bone Marrow Transplant Unit, Cancer Care Center, Augusta Victoria Hospital, Jerusalem, State of Palestine; c Dean SOM Al-Quds University Faculty of Medicine, Jerusalem, State of Palestine

**Keywords:** CML, TBI, Transverse myelitis, Rare, Case report

## Abstract

**Background:**

Transverse myelitis is a rare spinal cord inflammation with absence of a compression. It varies in presentation based on the pathology location, and mainly causes a combined deficit of motor, sensory, and autonomic functions. History, physical examination, and other diagnostic tests including blood tests and an MRI are important tools to establish a diagnosis.

A thorough neurological evaluation helps localize the affected region of the spinal cord. The management includes rehabilitation as any other spinal cord injury. If very severe, a multidisciplinary rehabilitation program will be required.

**Presentation:**

We explain a case in which a 43-year-old male patient, known to have chronic myelogenous leukemia (CML), on Imatinib (a tyrosine kinase inhibitor), started complaining of back pain at the level of the 10th rib. Different tests were made including a PET-CT (Positron Emission Tomography-Computed Tomography) which showed hypermetabolic bony lytic lesion in the left mandible at the level of temporomandibular joint, destruction of the 10th rib, and no evidence of spinal cord compression. Other etiologies were excluded, making transverse myelitis due to radiation for the patient's CML on top of the differential diagnosis.

**Conclusion:**

A thorough physical examination and diagnostic tests are important tools to exclude other etiologies of complex neurological deficit in a patient with CML.

## Highlights

• Transverse myelitis can be a possible complication post-radiation for CML treatment.
• Careful history taking, meticulous physical examination, and investigations are necessary.
• Avoid delaying intervention once a diagnosis has been made to avoid neurological impairment.

## 1 Introduction

Total body irradiation (TBI) is becoming a common procedure for the treatment of many malignancies and the conditioning protocols of bone marrow transplantation (BMT). Hence, this procedure is used to get the deepest response to destroy neoplastic cells or residual neoplastic cells, clearing the host marrow to allow repopulation with donor marrow cells, and providing sufficient immunosuppression to avoid allograft rejection by immunologically active cells in the host [1,2,5].

A reduced dose of TBI has been used for CML effectively for lowering the morbidity associated with myeloablation; however, it is not a standard procedure, its use has been increasing popularity in the last decades [10].

Adverse effects for this procedure include nausea, vomiting, sweating and irritability as well as renal dysfunction, interstitial pneumonia, lung injury, cataract, osteosarcoma, and cystitis in rare cases [3,4,6]. There are very few cases of transverse myelitis following radiation and it is very difficult to diagnose since you have to exclude many other causes (such as rheumatological diseases, demyelinating diseases or infectious etiologies) before being able to say that this condition was due to radiation [4].

Transverse myelitis is a neurological syndrome caused by spinal cord inflammation which can involve all age groups. The annual number of cases with transverse myelitis estimated varies from 1 to 5 per million cases [5].

The work has been reported in line with the SCARE 2020 criteria [11].

## 2 Case presentation

A 43-year-old male patient, known case of chronic myelogenous leukemia (CML) since 2015 and treated with Imatinib, was in his usual state of health until December 2019 when he started to complain from dull aching pain on the back (in the right side at the level of the 10th rib).

The pain was aggravated when the patient was in a supine position and with movement and it was associated with a feverish sensation and chills, but he did not complain from night sweats, headache or any neurological manifestation.

**Fig. 1:**
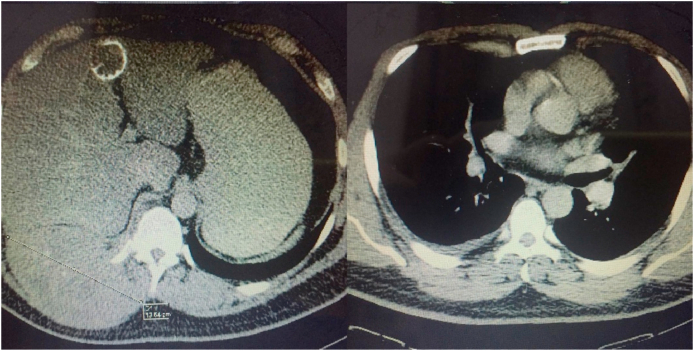
Whole CT-scan performed on 30^th^ December 2019 showing a calcified cystic structure.

A CT scan of the whole body was performed on 30^th^ December 2019 (for brain, neck, chest, abdomen, and pelvis) which showed a right postero-lateral heterogenous enhancing lesion with bone destruction of the 10th rib, measuring about 14 × 8 cm and with encasement to the right paraspinal muscle and the transversus process of the right 10th rib. Few fine calcifications and mild right sided pleural effusion with thickening of the right lower pleura were detected. A calcified cystic structure was also seen in the fourth segment of the liver, measuring 4 cm, suggesting a calcified hydatid cyst. (Fig. 1).

Furthermore, a tru-cut biopsy from the lesion was taken showing a poorly differentiated malignant neoplasm, mostly of hematolymphoid origin, that was consistent with a myeloid sarcoma (extramedullary myeloid tumor or chloroma).

After multiple disciplinary pathologist team, revision of the paraffine blocks showed large cells (mostly undifferentiated blasts), scattered with eosinophils and with reactive B and T lymphocytes (highlighted by CD20 and CD3). This neoplasm was positive for CD45, CD34, CD43, C-MYC and focally positive for C-Kit.

In January 2020, the patient underwent bone marrow trephine biopsy that showed slightly hypercellular trilineage bone marrow showing about 75% (instead of 60% that was the normal for his age). The megakaryocytes were moderately increased, some of them appeared immature and the reticulin showed +1 fibrosis.

A cytogenetic test was also performed, showing an abnormal karyotype with nine of the 20 analyzable cells examined, having t(9;22) which is known as Philadelphia translocation and seen usually in CML.

A real-time PCR for AML1-ETO fusion transcript resulted from t(8;21), for CBFB-MYH1 fusion transcript from inv(16) and for PML-RARa fusion transcript resulting from t(15;17) were not detected. A fragment length polymorphism for NPM1 mutation was also not detected, neither for FLT3 mutation.

However, the results for a real-time PCR for major and minor BCR-ABL fusion transcripts resulted from t(9;22) (q34;q11) were different since the major BCR-ABL fusion gene was detected but the minor gene was not.

A molecular analysis of TKIs mutation resistance was also performed in the same day, showing no specific mutations.

An immunophenotype showed a that the blasts were 3.4%, the monocytes were 4.3%, the granulocytes were 80% and the lymphocytes were 6.2%; this demonstrated that there was no evidence of excess blast percentage in the bone marrow sample.

A lumbar puncture was executed, and the CSF fluid was analyzed but was normal. CBC showed that the albumin was low (3.21 when the normal range is 3.7–5.2 g/dL), the calcium was slightly low (8.17 when the normal range is 8.6–10.3 mg/dL), the LDL was high (364 when the normal range is 135–225 u/L), the total protein was low (5.58 when the normal range is 6.6–8.7 g/dL), RBCs were low (3.83 when the normal range is 4.7–6.1 × 10^6^/μL), the hemoglobin was low (11.1 when the normal range is 13.5–18 g/dL) and the fibrinogen was high (618.38 when the normal range is 150–430 mg/dL).

A month later, a PET-CT scan was performed as a whole-body protocol. The images were acquired from the vertex to the mid-thigh showing:

• Head and neck: the included part of the brain demonstrated FDG metabolic activity in the cervical glandular tissue with no definite active focal lesion or hypermetabolic cervical lymph nodes.
• Chest: Evidence of right lower chest wall with mildly hypermetabolic soft tissue with underlying rib destruction involving the 10th rib with SUV max of 4.3 and associated with ipsilateral marked pleural effusion and a collapsed right lower lobe lung.
• Abdomen and pelvis: Physiologic limit of FDG metabolic activity in the liver (SUV max 3.5) as well as spleen and bowel with no evidence of active focal lesions. There was evidence of a right liver lobe calcified wall hydatid cyst.
• Musculoskeletal: Evidence of moderate hypermetabolic bony lytic lesion in the left mandible, at the level of the temporomandibular joint with SUV max 6.3. There was destruction of the 10th rib as mentioned above.

After that, the patient was started on Ponatinib (45 mg 1 × 1 po) and was intended to be enrolled in the bone marrow transplant program, so we had the plan to add to his medication the chemotherapy agent (7 + 3) but the patient denied the chemotherapy.

In June, a bone marrow trephine biopsy was taken, showing that there was no evolution from the last one. Real-time PCR for major BCR-ABL fusion transcript was done as a follow up, but it was not detected this time.

In August, a PET-CT scan was performed again and compared to the one that was performed in February. The head, neck, chest, abdomen, and pelvis had no evolution or regression; however, the hypermetabolic lesion in the left mandible was not appreciated anymore.

In September, the patient started to complain from shortness of breath, fatigability and low back pain radiated to the left lower limb with numbness. We performed an X-ray that showed right sided pleural effusion, so paracentesis was executed showing a mixture of inflammatory cells with many neutrophils but no evidence of malignancy. The WBCs were 178/μL with a lymphocyte percentage of 30% and granulocytes of 70%, the RBCs were 53000/μL, the LDH was 200 u/L and the fluid pH was 8 (normal range from 6.8 to 7.6). With this information, we concluded that the patient had a parapneumonic effusion that was exudative in origin, so we did a cytology analysis and a PCR for BCR-ABL, but the cytology was negative for malignant cells and the PCR was negative.

Also, a whole spine CT scan was performed, showing normal vertebral bodies height and alignment but no evidence of spinal cord compression. However, there was a huge bulge in the L5/S1 disc, so the patient underwent a surgical procedure (laminectomy) for management of the bulge.

One month later, the patient had worsening of the back pain and numbness, loss of sphincter tone and power in both lower limbs equally. A whole spine CT-scan was executed demonstrating an L5 left side laminectomy (due to the recent surgery) with soft tissue edema and thickening suspected to cause canal stenosis and diffuse disc bulges indicating thecal sac in L3-4 and L4-5.

Due to these findings, we started to think about the possibility that the patient could have transverse myelitis since he underwent radiotherapy for the treatment of his leukemia or due to viral infection causing this symptomatology.

In November, we carried out a lumbar puncture and analyzed the CSF. The analysis demonstrated that the protein was high (113.9 when the normal range is 15–45 mg/dL) and the WBCs were elevated (104 count/uL) with 25% of monocytes and 75% of polymorphonuclear leucocytes. It also showed dense neutrophilic infiltrate (hypercellularity) with no evidence of malignant cells seen or bacterial/fungal growth. A PCR for varicella zoster viral load in the CSF was also not detected, neither for JC virus, HSV-1 nor HSV-2.

We also tested the patient for anti-nuclear antibodies (ANA), anti-DNA antibodies and anti-Smith antibodies to rule out any rheumatological condition that could be the cause of this symptomatology but were all negative.

**Fig. 2:**
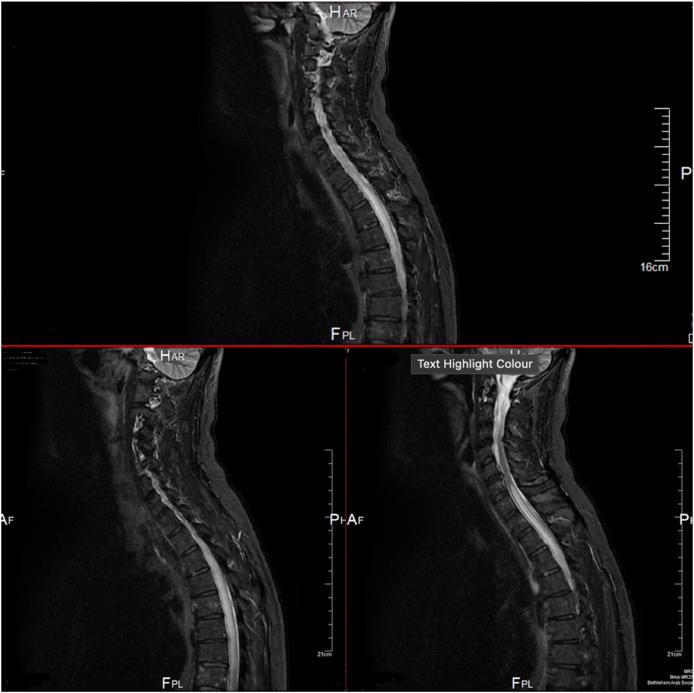
An MRI performed of 5^th^ November 2020 showing abnormal signal intensity within the thoracic cord extending from the mid T6 vertebral body level (superiorly) to the T11-T12 disc level inferiorly.

In terms of the clinical and laboratory findings, the patient was highly suspicious for transverse myelitis post-radiation, so he underwent MRI which showed that there was a region of abnormal signal intensity within the thoracic cord extending from the mid T6 vertebral body level (superiorly) to the T11-T12 disc level inferiorly associated with a patchy abnormal enhancement within the mid aspect of this lesion. There was a region of abnormal bone marrow signal intensity and apparent osteolysis involving the posterior medial aspect of the right 10th rib consistent with a malignancy at this site and small posterior disc herniations at multiple levels throughout the spine (Fig. 2).

At the end, and with all the laboratory and radiological findings, a diagnose of transverse myelitis post-radiation was on top in the differential diagnosis, so a pulse therapy with Solumedrol (1 g for five days) was started and the patient had a good partial response to this medication.

Plasmapheresis was also initiated, and the patient was sent to the rehabilitation center for further management.

## 3 Discussion

Transverse myelitis can result from a temporary demyelination caused by the radiation-related inhibition of oligodendroglial cells which produce myelin within spinal cord [5]. It is a rare condition, especially with the improvement in the delivery techniques, but reports of it have recently reemerged in the context of spine stereotactic body radiation therapy, or combination therapy with anticancer drugs (chemotherapy, targeted drugs, or immunotherapy) [7].

The vast majority of transverse myelitis cases are seen at 10–19 and 30–39 years of age; it has no familial tendency or gender predominance. Our case was a 43-year-old man without family history in terms of transverse myelitis.

Causes include bacterial and viral infections, vaccines, systemic autoimmune diseases (systemic lupus erythematosus, Sjogren's syndrome, sarcoidosis, and multiple sclerosis), paraneoplastic syndromes and spinal vascular events; although, there is large number of idiopathic cases [5].

Since radiation myelitis is not associated with any characteristic features, it can be considered only after all infectious, metastatic, neurologic, and orthopedic etiologies have been excluded. More specifically, opportunistic leptomeningitis (varicella zoster virus, cytomegalovirus, aspergillosis, tuberculosis, bacterial), epidural or vertebral metastasis, congenital or acquired spine malformations, preexisting central nervous system damage, and intracranial/spinal infarction secondary to an auto-immune vasculitic or hypercoagulable state must all be considered [8]. In our patient, there was no clinical evidence suggesting any of these causes. The patient's CSF serology and cultures showed no abnormal findings and confirmed no infectious pathogens.

It consists of a spectrum of clinical syndromes which typically develop 2–15 months following spinal cord exposure to therapeutic radiation. Self-limited presentations of minimal consequence, best typified by Lhermitte's sign (‘shocks’ along the spine or tingling in the hands secondary to head flexion), are usually seen within 4 months of treatment. More serious complications usually develop 9–24 months after treatment and are thought most likely to represent intramedullary infarction or bleeding due to vascular damage [7]. Although severe progressive myelitis is rare, it may potentially lead to catastrophic paresis and sensory loss [8].

Cerebrospinal fluid analysis of the lumbar puncture may either be normal or characterized by an increase in protein concentration. Contrast-enhanced MR imaging is frequently used as a diagnostic tool to help develop a differential diagnosis. Frequently described MR imaging include spinal cord expansion, atrophy, hyperintense signal changes on T2-weighted images, and contrast enhancement. However, these imaging findings are non-specific and can vary depending on the timing of MR imaging respecting the radiation exposure. Findings of swelling of the spinal cord, abnormal MR signal intensity, and intra-medullary contrast enhancement are similar to those seen in myelitis of other etiology [9].

Management depends on the cause; however, steroid therapy is recommended in idiopathic cases. The resolution may never occur, or it may be either partial or complete and it usually begins at months one and three [5]. Our patient was given a pulse therapy of Solumedrol and showed partial improvement. Plasmapheresis was initiated and referral to a rehabilitation center was recommended.

## 4 Conclusion

Acute transverse myelitis is more common than the chronic form since the symptoms are more detectable; however, taking into consideration this post-radiation complication and a prompt diagnosis of this condition with the appropriate management can help in the prevention of severe neurological impairments.

Although it is a rare condition, we recommend physicians to consider transverse myelitis when listing their differentials for neurological dysfunction complaints in CML patients who had radiation therapy.

## Sources of funding

This research did not receive any specific grant from funding agencies in the public, commercial, or not-for-profit sectors.

## Ethical approval

The study is exempt from ethical approval in our institution.

## Consent

Written informed consent was obtained from the patient for publication of this case report and accompanying images. A copy of the written consent is available for review by the Editor-in-Chief of this journal on request.

## Authors’ contributions

Study concept or design: Hamdi Al-Janazreh. Writing the manuscript: Yousef S. Abuzneid, Motasem Haif, Shahd T. Idais, Baraa Asakrah, Sufana M. Ajwa, Shifa Sarahneh and Hani Abdeen. Review & editing the manuscript: Yousef S. Abuzneid and Hamdi Al-Janazreh.

## Declaration of competing interest

There is no conflict of interest.
